# Physiotherapy for endometriosis-associated pelvic pain: a systematic review and meta-analysis

**DOI:** 10.1093/pm/pnaf083

**Published:** 2025-07-24

**Authors:** Gökçe Can, Isabel Pinto Amorim das Virgens, Boglárka Fehér, Enikő Pálma Orbán, Péter Fehérvári, Ferenc Bánhidy, Péter Hegyi, Ágnes Andrea Mayer, Nándor Ács

**Affiliations:** Centre for Translational Medicine, Semmelweis University, 1085 Budapest, Hungary; Centre for Translational Medicine, Semmelweis University, 1085 Budapest, Hungary; Centre for Translational Medicine, Semmelweis University, 1085 Budapest, Hungary; Department of Obstetrics and Gynecology, Semmelweis University, 1082 Budapest, Hungary; Centre for Translational Medicine, Semmelweis University, 1085 Budapest, Hungary; Department of Physiotherapy, Faculty of Health Sciences, Semmelweis University, 1088 Budapest, Hungary; Centre for Translational Medicine, Semmelweis University, 1085 Budapest, Hungary; Department of Biostatistics, University of Veterinary Medicine, 1078 Budapest, Hungary; Centre for Translational Medicine, Semmelweis University, 1085 Budapest, Hungary; Department of Obstetrics and Gynecology, Semmelweis University, 1082 Budapest, Hungary; Centre for Translational Medicine, Semmelweis University, 1085 Budapest, Hungary; Institute for Translational Medicine, Medical School, University of Pécs, 7624 Pécs, Hungary; Institute of Pancreatic Diseases, Semmelweis University, 1083 Budapest, Hungary; Centre for Translational Medicine, Semmelweis University, 1085 Budapest, Hungary; Department of Physiotherapy, Faculty of Health Sciences, Semmelweis University, 1088 Budapest, Hungary; Centre for Translational Medicine, Semmelweis University, 1085 Budapest, Hungary; Department of Obstetrics and Gynecology, Semmelweis University, 1082 Budapest, Hungary

**Keywords:** chronic pelvic pain, electrotherapy, endometriosis, exercise, manual therapy, physiotherapy, physiotherapy modalities

## Abstract

**Importance:**

Endometriosis is a disease often associated with chronic pelvic pain. It affects around 190 million females of reproductive age globally.

**Objective:**

To investigate the effect of physiotherapy techniques (PTs) in relieving endometriosis-associated pain.

**Design:**

This systematic review and meta-analysis followed the PRISMA 2020 and Cochrane Handbook guidelines. The study protocol was followed and registered on PROSPERO (CRD42023474231).

**Setting:**

A systematic search was conducted in 3 databases: PubMed, Embase, and Cochrane.

**Participants:**

We used the following PICO strategy: population, women with endometriosis-associated pelvic pain; intervention, PTs; comparator, non-PTs; outcome, pelvic pain changes. The article selection was conducted by 2 independent reviewers.

**Main Outcome(s) and Measure(s):**

Two authors extracted data independently from the eligible articles. For continuous outcomes, the mean difference (MD) in change scores between intervention and control groups was used as an effect size with a 95% confidence interval (CI). Within-group correlation of before- and after-treatment was assumed to be equal across groups and studies.

**Results:**

Out of 8 eligible studies identified in our selection, 7 were included in the quantitative analysis. PTs were effective in reducing pain compared to non-PTs (MD −1.97, CI −2.99 to −0.95), and physiotherapy modalities (electrotherapy and laser devices) had the greatest reduction in pain levels (MD −2.03, CI −3.9 to −0.14) among all studies. Additionally, locally applied techniques resulted in greater pain reduction than the generally applied techniques.

**Conclusion and Relevance:**

Physiotherapy techniques are effective in reducing pain in women with endometriosis, especially when applied locally.

## Teaser

Endometriosis is a disease often associated with chronic pelvic pain. We conduct this systematic review and meta-analysis to investigate the effect of physiotherapy techniques in relieving endometriosis-associated pain by following the PRISMA 2020 guideline and the Cochrane Handbook. Seven of eight eligible studies identified in our selection were included in the quantitative analysis. The results of this study suggest that physiotherapy techniques are effective in reducing pelvic pain in women with endometriosis, compared to non-physiotherapy techniques.

## Introduction

Endometriosis is a chronic inflammatory condition marked by the growth of endometrial tissue located outside the uterus.^1^ It can lead to a wide range of symptoms and complications and is often associated with infertility, pelvic pain, dyspareunia, or dysmenorrhea.^1^^,^^2^ It affects roughly 10% of women of reproductive age globally.^3–5^ This would correspond to over 200 million women, according to the World Bank population estimates for 2023.^5^^,^^6^ Almost 1 in 2 women with endometriosis experience chronic pelvic pain (CPP).^7^ Moreover, 70% of them experience pain during menstruation, ranging in severity from mild to severe.^7^ Hence, quality of life (QoL) is severely affected by this pain, and treating women with endometriosis empowers those affected by it by affirming their right to have optimal QoL, sexual health, and overall well-being.^3^^,^^8^

Some of the current treatment methods for endometriosis-associated pelvic pain (EAPP) focus on pharmacotherapy, particularly the prescription of non-steroidal anti-inflammatory drugs (NSAIDs) and analgesics (painkillers).^3^^,^^9^^,^^10^ Despite their effectiveness, long-term use of these medications is known for their range of side effects, from gastric problems to cardiovascular complications.^1^^,^^11–13^ Moreover, they are not suitable for individuals with contraindications.^12^^,^^13^ These may lead patients to refuse this treatment method and look for alternative options.

One of the recent clinical guidelines for endometriosis emphasizes that more research is needed to expand therapeutic options and find alternative remedies to standard care.^1^ Another updated guideline on CPP recommends employing a holistic management approach that encompasses the evaluation of pharmacological and non-pharmacological treatment methods and the active involvement of patients.^14^

Therefore, physiotherapy techniques (PTs), with their anti-inflammatory and analgesic effects, may be beneficial in the treatment of endometriosis-related pelvic pain.^7^^,^^15^ In other health conditions (ie, temporomandibular joint myofascial pain, knee osteoarthrosis, or low back pain), several studies have already shown that physiotherapy, or usual care supplemented with physiotherapy, is more cost-effective than usual care and has fewer side effects.^16^^,^^17^ However, cost-effectiveness might be subjective and may depend on the chosen treatment techniques, number of sessions, and insurance policies of the countries.

Although the pain-relieving potential of PTs is known, there is no meta-analysis about endometriosis-related pelvic pain focusing exclusively on physiotherapy methods to the best of our knowledge. The effectiveness varies significantly across diverse PTs, emphasizing the need for further research. This condition makes it challenging to propose an evidence-based alternative method for pain management in clinical practice. It highlights the need to explore the potential of physiotherapy and investigate its effectiveness in greater detail. Therefore, in this systematic review and meta-analysis, we aimed to evaluate the efficacy of various PTs in alleviating endometriosis-related pelvic pain.

## Method

We report our systematic review and meta-analysis based on the recommendation of the PRISMA 2020 guideline^18^ (see Table S4) and followed the Cochrane Handbook.^19^ The study protocol was registered on PROSPERO (registration number CRD42023474231), and we adhered to it. No amendments were made to the protocol.

### Eligibility criteria

We used the following PICO strategy: Population, women who suffer from endometriosis with pelvic pain; intervention, PTs; comparator, non-PTs; and outcome, changes in pelvic pain.

Inclusion criteria were (a) application of various PTs, (b) being a female diagnosed with endometriosis experiencing pelvic pain, (c) use of the 0-to-10-point pain measurement scales (ie, Visual Analog Scale [VAS], Numerical Rating Scale [NRS]), or an equivalent pain measurement scale and questionnaires to assess pain before and after treatments. We excluded the studies that included (a) non-conventional PTs and interventions other than physiotherapy (yoga, Pilates, acupuncture, etc), (b) populations with diseases other than endometriosis, (c) studies involving animal or in vitro studies, (d) studies that do not report pain as an outcome, (e) females who do not experience pelvic pain associated with endometriosis, and (f) we excluded studies that did not use the pain measurement scales mentioned to assess outcome.

Seven of 8 studies used 0-to-10-point pain measurement scales to assess pain with VAS and NRS, while one study used the Present Pain Intensity Scale (PPIS), a pain measurement scale ranging from 0 to 4. Our inclusion criteria were to include studies with 0-to-10-point pain measurement scales. Therefore, to provide a fair comparison, the measurement scores of the study with PPIS were calculated by multiplying the values by 1.5, as suggested by statisticians. In this way, they become similar to the 0-to-10-point measurement scales.

To classify the interventions by similarity, studies were divided into 3 categories: (1) physiotherapy modalities (PM), which involve methods that use energy to deliver treatment using a device (electrotherapy techniques: Transcutaneous electrical nerve Stimulation [TENS], Transcranial direct current stimulation, Neuromuscular electrical stimulation [NMES], and laser therapy); (2) exercises that included methods using various movements as exercise (multimodal supervised therapeutic exercise program); and (3) manual therapy (MT) comprising methods that used hands primarily as Thiele massage and a MT protocol. Comparison groups were defined as no treatment, placebo, or any other non-physiotherapeutic technique. Detailed comparison groups for each study can be found in Table 1.

**Table 1.** Basic characteristics of the studies with main outcomes.

Study	Study design	Intervention group	Intervention type	Comparison type	Intervention period	Application area	Pain scale
Artacho et al. 2023 (MA)	Randomized controlled trial	Exercise/Activity	Multimodal exercise	Control	9 weeks	General and local	Numerical Rating Scale
Del Forno et al. 2021 (MA)	Randomized controlled trial	Manual therapy	Thiele massage	Control	11 weeks	Local	Numerical Rating Scale
Gomez et al. 2023 (MA)	Randomized controlled trial	Manual therapy	A manual therapy protocol consisting of the combination of soft tissue and articulatory techniques	Placebo	8 weeks	Local (additional regions)	Visual Analog Scale
Lutfi et al. 2023 (SR)	Randomized controlled trial	Exercise/Activity	I1: Telehealth-delivered exercise group, I2: VR-delivered exercise group	Control	1 hour	General	Visual Analog Scale
Mechsner et al. 2023 (MA)	Randomized controlled trial	Advanced physiotherapy methods	Electrotherapy (transcranial direct current stimulation)	Placebo	2 weeks	General	Numerical Rating Scale
Mira et al. 2020 (MA)	Randomized controlled trial	Advanced physiotherapy methods	Electrotherapy (hormonal treatment plus self-applied TENS)	Control (only hormonal treatment)	8 weeks	Local	Visual Analog Scale
Thabet et al. 2018 (MA)	Randomized controlled trial	Advanced physiotherapy methods	Pulsed high-intensity laser therapy	Placebo	8 weeks	Local (additional regions)	Present Pain Intensity Scale
Bi et al. 2018 (MA)	Non-RCT, retrospective study	Advanced physiotherapy methods	Electrotherapy (NMES)	Control	10 weeks	Local (additional regions)	Numerical Rating Scale

Abbreviations: MA, meta-analysis; SR, systematic review.

### Information sources and search strategy

Our systematic search was conducted on November 14, 2023. To maintain the methodological integrity of our study and provide a more comprehensive and reliable diversity of data, we used 3 databases: PubMed, Embase, and Cochrane. The search key was based on 2 domains: population (women with endometriosis) and intervention types (PTs). The detailed search keys and strategies are available in the Supplementary Material (see Table S5). No filters or restrictions were applied for the language and publication date of the study.

### Selection process

To remove duplicates, we imported the selected articles into EndNote 20,^20^ which were then detected by using automatic and manual exclusion tools. Moreover, the remaining articles were exported to Rayyan.^21^ The selection was conducted by 2 independent reviewers (GC and BF) for title, abstract, and full text. Disagreements were resolved by a third investigator (E.P.O.). Cohen's Kappa coefficient was calculated for interrater reliability. After the search process was completed, the Citation Chaser tool^22^ was used to identify additional articles in the reference lists. Moreover, additional libraries were searched, and emails were sent to the authors of full texts not found to retrieve further articles.

### Data extraction

Data were extracted independently from eligible articles by 2 authors (G.C. and B.F.) using Microsoft Excel to import the data. Disagreements were resolved by a third investigator (E.P.O.).

### Data items

The following data were extracted: Name of first author, publication year, DOI number, study design, study population and characteristics, intervention type, study duration, pain assessment scales, application surface for intervention, age, medication consumption, and other relevant variables.

### Study risk of bias assessment and GRADE

Two authors independently assessed the risk of bias (RoB) using the Cochrane tools: RoB-2 for randomized controlled trials (RCTs) and ROBINS-I for non-randomized studies. The quality of studies included was evaluated using the RoB score defined in the Cochrane Handbook for Systematic Reviews of Interventions. This score includes criteria such as random sequence generation, blinding of participants and investigators, blinding of outcome assessment, selective reporting, allocation concealment, and other biases. Finally, all studies were categorized as having a low, high, or unclear RoB, as recommended in the assessment guide.

Subsequently, the GRADE assessment was conducted by 2 independent researchers, and disagreements were resolved through mutual agreement between the 2 researchers (G.C. and E.P.O.). Final decisions were made between the 4 levels of the GRADE assessment tool as follows: high, moderate, low, and very low.

### Synthesis methods

Both qualitative and quantitative synthesis of the data was performed. Statistical analyses were conducted using R version 4.3.2 (R Core Team, 2021).^23^ Meta-analyses for computations and graphical representation were performed with the help of the meta package (Schwarzer, 2022, version 7.0.0)^24^ and the dmetar package (Cuijpers et al., 2022, version 0.1.0).^25^ The minimum number of studies to conduct a meta-analysis was 3.

For continuous outcomes, the mean difference (MD) between change scores in the intervention and control groups was used as an effect size measure with a 95% confidence interval (CI). If change scores (change in pain score between pre- and post-treatment compared to baseline) could not be extracted, they were calculated using baseline and post-treatment values. Within-group correlation of before- and after-treatment was assumed equal across groups and studies.

The random effects model was chosen for the meta-analyses. Adjustments to the CIs were made using the Hartung–Knapp method.^26^^,^^27^ The τ^2^ estimate utilized the Paule–Mandel method, and the CI for τ^2^ was determined using the Q profile method.^28^

Statistical heterogeneity was assessed using the Cochrane Q test and I^2^ values, where statistical significance was considered if *P* < .1.^29^ Publication bias was examined via a funnel plot with the logarithm of effect size against the standard error for each trial.

In addition, outlier and influence analyses were conducted following the methodologies proposed by Harrer et al.^28^ and Viechtbauer and Cheung.^30^

## Results

### Search and selection

**Figure 1.**
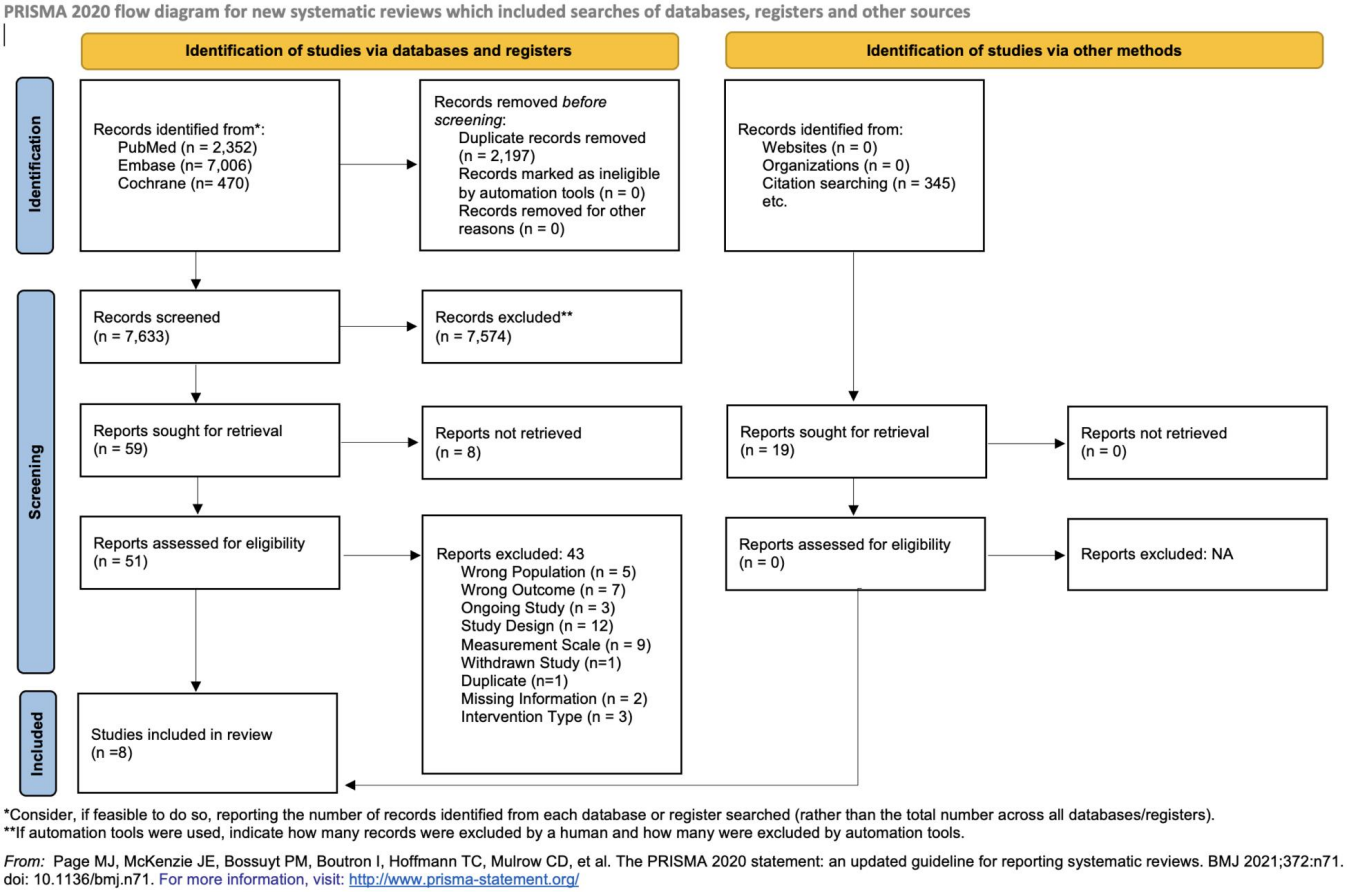
PRISMA 2020 flowchart representing the study selection process.

Of 9828 articles, 8 studies (7 RCTs and a retrospective study) were eligible to be included in our study. Of these, 7 were suitable to be pooled together for meta-analysis. The total sample size was 433, ranging from 30 to 154 participants among studies. Details about the selection process can be found in the PRISMA Flowchart (Figure 1).

### Basic characteristics of studies included

Baseline characteristics, demographic characteristics, and the pelvic pain values of the studies are detailed in the Supplementary Material (see Tables S1-S3). All intervention and control groups were similar at baseline in each study, including baseline pain scores (see Figure S2), and the changes between these groups can be seen in Figure S2a. Additionally, the time-effect relationship is presented in Figure S1.

### Intervention categories and types

**Figure 2.**
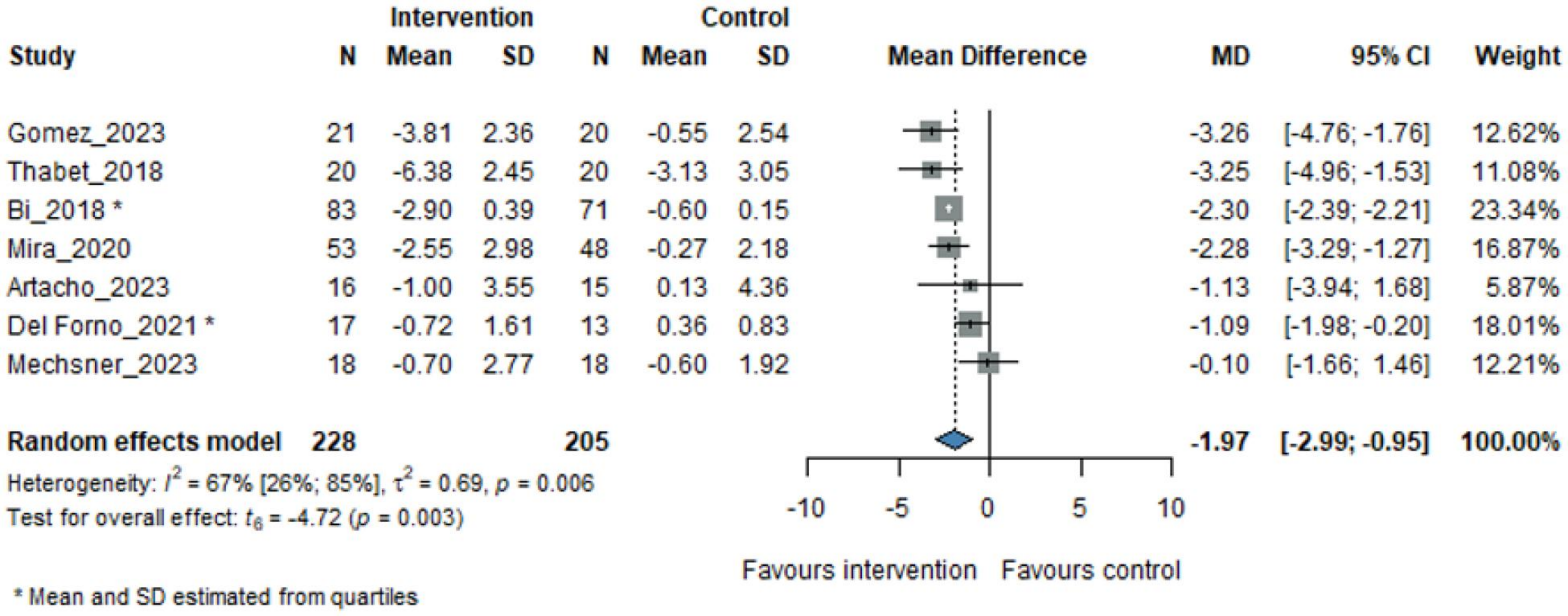
Pain change from baseline to the longest follow-up time.

Our analyses demonstrated that, regardless of the specific method used, PTs were more effective in reducing pain than non-PTs, resulting in a reduction of approximately 2 points on the pain scale (MD −1.97, 95% CI −2.99 to −0.95; Figure 2).

**Figure 3.**
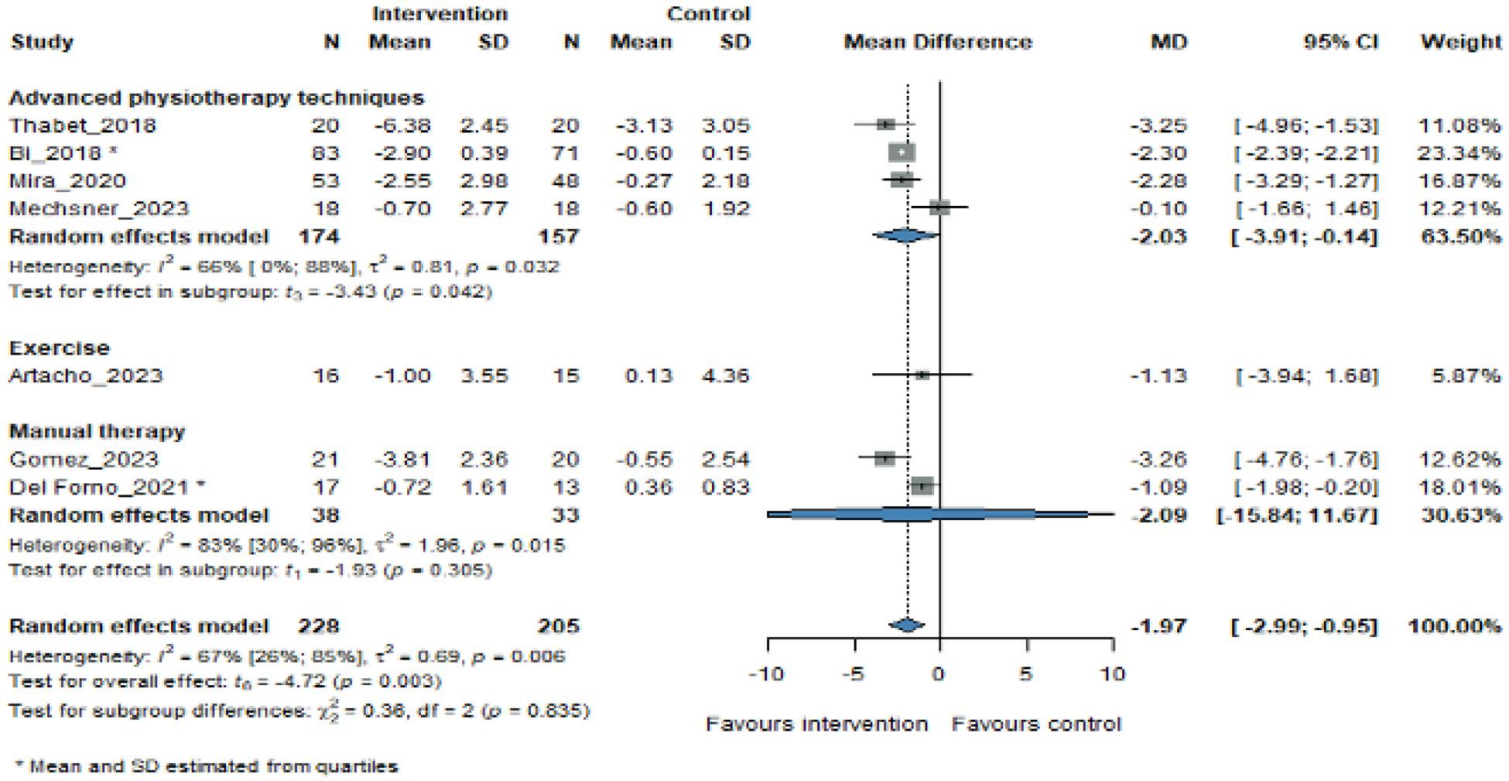
Effect of intervention types on pain change from baseline to the longest follow-up time.

Out of the 3 physiotherapy categories, PM were the most effective techniques (MD −2.03, 95% CI −3.91 to −0.14, *P *= .042; Figure 3) in reducing pelvic pain. Therefore, there was not enough data for the exercise and MT groups to reinforce this finding.

Bi et al.,^31^ Mira et al.,^32^ Mechsner et al.,^33^ and Thabet et al.^34^ used PM to relieve pain in patients. Techniques such as TENS, transcranial electrotherapy, and laser were used in the latter, and a significant difference in pain change was observed between the intervention and control groups in all studies. Although found to be effective in reducing pelvic pain, PM was the only method that mentioned the availability of side effects among all physiotherapy methods. These side effects ranged from headache to muscle spasms, occurring only during electrotherapy interventions, and were not reported in the study on laser therapy.^34^ Detailed information on side effects can be found in the Supplementary Material (Table S2). Among the studies in the PM category, the laser applications method (Mechsner et al.^33^) provided a greater efficacy (MD −3.25, 95% CI −4.96 to −1.53) in reducing pelvic pain due to endometriosis without significant adverse effects.

Our meta-analysis included 2 studies that used MT techniques. Gomez et al.^35^ applied a MT protocol consisting of a combination of soft tissue and articulatory techniques, whereas Del Forno et al.^36^ performed an intervention with the Thiele massage. Both interventions were successful in reducing pain (MD −2.09, 95% CI −15.84 to 11.67, *P *= .305) compared to control groups and yielded not statistically but clinically significant results. However, there were not enough studies to determine a definite conclusion with statistical significance.

Two of the studies on exercise were deemed suitable to be included in our study. Lutfi et al.^37^ compared the 2 intervention groups (telehealth-delivered exercise and Virtual Reality [VR]-delivered exercise) with the control group and found that the exercise groups were more beneficial than the control group. A study by Artacho et al.^38^ was the only study with an exercise intervention in our meta-analysis. The study showed that exercise was effective in reducing pelvic pain (MD −1.13, 95% CI −3.94 to 1.68) compared to the control group. However, compared to other categories, it may be less effective than PM and MT techniques. It is also good to mention that more research is needed regarding the effects of exercise on endometriosis-related pelvic pain in larger sample sizes to make a clear conclusion.

### Effect of application site on pelvic pain

**Figure 4.**
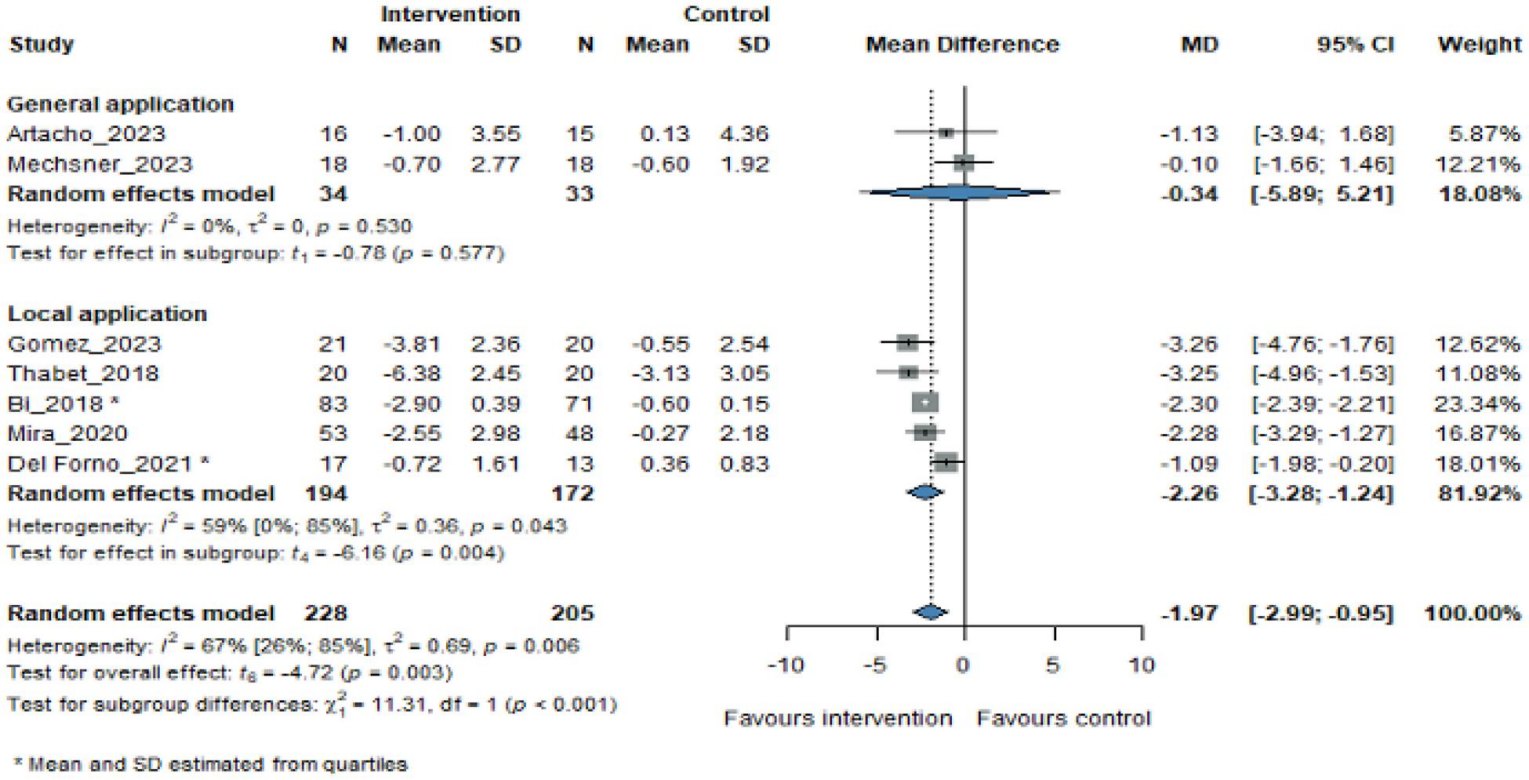
Effect of application sites on pain change from baseline to the longest follow-up time.

Of the 7 studies, 5 used locally applied interventions and 2 used generally applied interventions to relieve pain in patients. Our meta-analysis results highlight that locally applied interventions might be more effective (MD −2.26, 95% CI −3.28 to −1.24, *P *= .004; Figure 4) in the management of pelvic pain in women with endometriosis, while emphasizing the need for more studies with the generally applied techniques.

### Risk of bias and GRADE assessments

We found an overall moderate RoB in all studies. Detailed results of the analyses can be found in the Supplementary Material (see Figures S3-S5).

Subsequently, the final decisions of the GRADE assessment were made at the 4 levels of the tool. It resulted in low-quality evidence. The detailed results of the analyses can be found in the Supplementary Material (see Figure S6).

## Discussion

We aimed to evaluate the efficacy of PTs in alleviating endometriosis-related pelvic pain by synthesizing evidence from multiple studies. The findings of our study underscore a clearer understanding of how physiotherapy can be integrated into the management of endometriosis and which methods are most effective for improving patient outcomes. Our review focused on exercise, MT, and PM, revealing that these non-invasive techniques can be valuable in the management of endometriosis-related pelvic pain.

The effectiveness of physiotherapy treatments was one of the primary outcomes of this review. Our study demonstrates that PTs are superior to comparison groups in relieving EAPP across all studies included in our analysis.

To the best of our knowledge, there were only 2 similar studies on the topic. In their systematic review and meta-analysis, Abril-Coello et al.^8^ investigated the effectiveness of conservative, non-invasive, and non-pharmacological methods compared to sham, placebo, and control. The study included 6 RCTs, 3 of which included physiotherapy treatments (exercise, laser therapy, and electrotherapy)—the remaining studies reported on other interventions, such as yoga and patient education. In the study, Abril-Coello et al. suggested that non-pharmacological conservative therapies offer an alternative therapy option for patients with endometriosis. Although our results align with theirs, our study differs in that we focused only on physiotherapy treatment, emphasizing our expertise in the field and pursuing a more specialized approach rather than including other conservative therapy options.

Furthermore, the increasing number of studies has allowed us to include a wider range of PTs. Another feature of our study is the clinical relevance of our findings, with a change in pain of nearly 2 points.

Another study on the topic was conducted by Mira et al.^39^ They investigated the effect of complementary treatments on patients with endometriosis. In their review, Mira et al.^39^ included studies on PTs, such as exercise and electrotherapy, as well as relevant techniques other than physiotherapy, such as yoga and acupuncture. They concluded that complementary treatments were effective in alleviating endometriosis-associated pain. Still, not all methods were superior to the comparison groups.

Mira et al.^39^ also emphasized that acupuncture was the most beneficial technique of these interventions. However, professional standards for physiotherapy vary between countries,^40^ and acupuncture is not listed among the interventions that can be applied according to the World Confederation for Physical Therapy policy statement.^41^ This may raise doubts as to whether acupuncture is a valid physiotherapy method. So, we have decided to exclude this treatment method from our study. Hence, although their research demonstrates the benefits of complementary treatment options, more substantial evidence is needed on the optimal physiotherapy intervention for women with endometriosis.

On the other hand, our study highlights an additional research area that demands further investigation. Of the studies included, PM (−2.03, *P *= .042) demonstrated superior efficacy compared to other physiotherapy methods (exercise [−1.13] and MT [−2.09, *P *= .305]). However, only one study on exercise therapy was included in the meta-analyses, which indicates a need for more studies on exercise. Lutfi et al.^37^ included 19 participants in their study, and due to the implementation of a single 1-hour intervention session, this was not included in the meta-analysis. Nevertheless, it is important to add that exercises via VR and telehealth are also beneficial in endometriosis-related pelvic pain, according to the results of their study.

Another important point to discuss would be the heterogeneity of the study. The lack of the number of studies in the existing literature on physiotherapy and its effects on endometriosis made it difficult to conduct a meta-analysis on this topic. Therefore, similar to previous studies, our study has presented moderate to high heterogeneity. In order to make the study feasible and increase the number of studies to be analyzed, while other studies have included different conservative treatment methods, we chose to evaluate the existing physiotherapy models by dividing them into categories with similar functions. Although this may reduce the reliability of our study, the effort to present the strongest possible evidence within the scope of existing studies has shed light on the existence of different options that can be used in clinical treatment and the necessity for further research.

In addition, physiotherapy approaches address the functional limitations and impairments of patients with endometriosis.^41^ The benefits of physiotherapy treatments can be directed at improving mobility, QoL, and overall health outcomes.^41^ As changes in pain levels might be directly related to improvements in the QoL of patients, we focused on outcomes that exactly corresponded to pain. However, Abril-Coello et al.^8^ and some of the studies included in our review also investigated the QoL results as outcomes. In a study by Abril-Coello et al.,^8^ 5 studies assessed QoL and found significant outcomes only for the sub-variable physical function.

In our review, 5 of the 8 studies (Artacho et al.,^38^ Mira et al.,^32^ Gomez et al.,^35^ Thabet et al.,^34^ and Bi et al.^31^) investigated QoL measurements. They all reported that physiotherapy interventions were beneficial for improving QoL. However, Bi et al.^31^ noted that significant results were visible after 10 weeks of treatment.

In distinction from other research in the field, the result of our analysis highlights the efficacy levels of the localization of techniques. Even though there are not enough number of the study to compare application sites with clear evidence. Our study suggests that locally applied techniques, such as massage or MT protocols, may be more effective in relieving pain by targeting areas affected by endometriosis, and it is worth doing more research to understand the mechanism and efficacy behind it.

### Strengths and limitations

Therefore, several limitations must also be acknowledged. Our study included a relatively small number of cases and had a limited sample size, which may have influenced the generalizability of the findings. Additionally, the short intervention periods in some of the studies prevented us from observing the long-term effects of the interventions. In addition, instead of using only 0-10 pain measurement scales, we included one study that used a 0-4 scale. We converted the 0-4 values to match the 0-10 scale to ensure consistency. Still, this adjustment can be considered as another limitation of the study.

Furthermore, due to the lack of information in most studies, uncontrolled comorbidities associated with medication intake were another limitation. Also, the inclusion of studies with subjective assessment methods (eg, VAS) influenced the results of our RoB analyses. Due to the tool’s structure, some studies directly considered a high RoB, regardless of the results in other domains. However, as other parameters were mostly considered to have a low RoB, it should be noted that these results do not truly reflect the real overall RoB of studies in our meta-analysis. Lastly, the inclusion of one non-randomized study affects the results of the GRADE assessment. Accordingly, we had to start the evaluation with a low level of evidence. However, it is essential to note that 6 of the 7 studies included in our meta-analysis were RCTs.

For the strengths of our analysis, we adhered to our pre-registered protocol. To ensure fairness, we conducted both univariate and multivariate analyses and applied a rigorous methodology. Notably, our study included only recognized physiotherapy methods and highlighted the effectiveness of the localization of techniques used. In addition, the inclusion of mostly RCTs further strengthens our study. This study helps to achieve optimal outcomes in clinical practice and enhance treatment efficacy. Moreover, our research addresses and fills a significant gap in the literature on endometriosis-related pain by incorporating updated studies from 2018 to 2023.

To the best of our knowledge, this systematic review and meta-analysis is the only study that investigates the effectiveness of localization of the techniques applied, focusing exclusively on PTs. However, although this study provides insight into the localization, it should be noted that there was not enough data for a meaningful comparison.

This review underscores the potential of physiotherapy as an effective intervention to reduce EAPP. It provides valuable insights for clinicians, researchers, and authors by offering clinically relevant and statistically significant results.

### Implications for practice and research

We strongly recommend that clinicians include PTs in their therapeutic options. By integrating physiotherapy into standard treatment protocols, healthcare providers can offer a more comprehensive and patient-centered holistic approach to managing endometriosis. **More specifically, to ease women’s access to treatment,** PM such as laser and electrotherapy devices should be available in the endometriosis and pelvic therapy units. **To help women with endometriosis manage their pain independently and increase their autonomy,** classical massage techniques, pelvic floor muscle stretching methods, and exercises that can be performed at home should also be prescribed by doctors and physiotherapists in addition to medication. **To reduce the possibility of dependence on painkillers and potential side effects,** the consumption of painkillers should be restricted if PTs are found to relieve pain.

Additionally, we highly recommend that insurance companies cover physiotherapy for women with endometriosis to increase the accessibility and affordability of treatments. Lastly, more research is needed to understand the effectiveness of physiotherapy with larger sample sizes and with or without additional drugs to improve recommendations on treatment options for those with endometriosis-associated pain. There is also a need for studies with longer follow-up times to have an idea about the continued effects of the studies. In addition, it is imperative to understand the most beneficial physiotherapeutic intervention. Thus, translation of research into clinical practice may improve the well-being of patients with endometriosis.^42^^,^^43^

## Conclusion

The result of this study highlights the positive effects of physiotherapy on women with endometriosis. In this regard, our study has developed the view that PTs might be effective in reducing pelvic pain in patients with endometriosis, especially when applied locally. Of the various techniques, PM were found to be more effective in reducing pain. Given its non-invasive nature and the wide range of applicable techniques, physiotherapy offers a valuable area for further research, particularly for women who experience contraindications or adverse effects from available pain management methods, thereby helping to enhance more precise clinical treatment recommendations.

## Supplementary Material

pnaf083_Supplementary_Data

## Data Availability

All primary data presented in this article can be found in the original texts of the studies.
